# Automated imaging coupled with AI-powered analysis accelerates the assessment of plant resistance to *Tetranychus urticae*

**DOI:** 10.1038/s41598-024-58249-7

**Published:** 2024-04-05

**Authors:** Ewelina Złotkowska, Anna Wlazło, Małgorzata Kiełkiewicz, Krzysztof Misztal, Paulina Dziosa, Krzysztof Soja, Anna Barczak-Brzyżek, Marcin Filipecki

**Affiliations:** 1 Department of Plant Genetics, Breeding and Biotechnology, Institute of Biology, Warsaw University of Life Sciences, Warsaw, Poland; 2 Department of Applied Entomology, Institute of Horticultural Sciences, Warsaw University of Life Sciences, Warsaw, Poland; 3 Faculty of Mathematics and Computer Science, Jagiellonian University, Kraków, Poland; 4 diCELLa Ltd., Kraków, Poland

**Keywords:** Plant stress responses, Entomology, Behavioural methods, High-throughput screening

## Abstract

The two-spotted spider mite (TSSM), *Tetranychus urticae,* is among the most destructive piercing-sucking herbivores, infesting more than 1100 plant species, including numerous greenhouse and open-field crops of significant economic importance. Its prolific fecundity and short life cycle contribute to the development of resistance to pesticides. However, effective resistance loci in plants are still unknown. To advance research on plant-mite interactions and identify genes contributing to plant immunity against TSSM, efficient methods are required to screen large, genetically diverse populations. In this study, we propose an analytical pipeline utilizing high-resolution imaging of infested leaves and an artificial intelligence-based computer program, MITESPOTTER, for the precise analysis of plant susceptibility. Our system accurately identifies and quantifies eggs, feces and damaged areas on leaves without expert intervention. Evaluation of 14 TSSM-infested *Arabidopsis thaliana* ecotypes originating from diverse global locations revealed significant variations in symptom quantity and distribution across leaf surfaces. This analytical pipeline can be adapted to various pest and host species, facilitating diverse experiments with large specimen numbers, including screening mutagenized plant populations or phenotyping polymorphic plant populations for genetic association studies. We anticipate that such methods will expedite the identification of loci crucial for breeding TSSM-resistant plants.

## Introduction

It is well known that herbivorous pest fitness traits are differently affected by host plants; and each trait variation may reflect distinct resistance or susceptibility mechanisms. Therefore, the classification of plant resistance should consider multiple traits, such as comparing the development of the pest population, reproductive potential and the severity of other symptoms^1^. These methods, however, require broad expertise on the given pest biology and behavior^2,3^. In addition, the difficulty and labor intensity increase the smaller the pest/symptom size, which necessitates the use of a microscope equipped with a high-resolution camera^4^. Thus, assessments of host plant resistance to a generalist such as the spider mite seem to be a serious bottleneck, especially when multiple pest variants and plant cultivars in diverse environmental conditions are to be compared at one time.

Our main object of interest is the phenomenon of natural host plant resistance to the two-spotted spider mite (TSSM; *Tetranychus urticae* Koch 1836, (*Acariformes*: *Trombidiformes*: *Tetranychidae*)). Its cosmopolitan and generalist nature—it feeds on over 1100 host plants^5,6^—together with its short lifespan, high female fecundity and efficient xenobiotic metabolism make TSSM one of the most significant agricultural pests globally. TSSM is a mesophyll cell-content feeder that uses stylet-like mouthparts formed by two cheliceral digits to inflict damage to spongy mesophyll, palisade parenchyma, and chloroplasts within the plant cell, eventually causing cell death and the appearance of chlorotic spots^7^. In optimal conditions (temp. 25–30 °C and 45–55% relative humidity), TSSM development (from egg to adult stage) can be completed in as little as seven days^8–10^. Such a short life cycle results in the emergence of up to 25 exponentially growing generations a year^11^. A mated female can lay up to 20–30 transparent eggs per day in optimal conditions (more than 200 over its lifespan), mostly on the abaxial side of a leaf^12–15^. Taken together, TSSMs can quickly propagate incidental mutations or inheritable epigenetic changes, giving them higher pesticide tolerance. Consequently, it has gained the dubious reputation of being the most resistant species to pesticides^16,17^.

For all these reasons, new mite-pest resistant crop cultivars are needed. This requires the identification of new loci and the characterization of underlying plant resistance mechanisms. To date, molecular approaches have yielded some promising results, but they have mainly focused on candidate gene selection based on their differential expression upon TSSM attack^18,19^. An alternative solution would be the screening of large polymorphic populations of host plant accessions and mapping chromosomal regions involved in resistance-related traits^20^. To apply such an approach, a high-throughput method that quantifies mite activity on the host plant is needed. Such a method should be unbiased (avoiding human-based arbitrary assessment), precise (measuring instead of rating) and efficient (relatively short time of data acquisition). Moreover, there are several measures of plant susceptibility/resistance, and they may be biased toward diverse resistance mechanisms,therefore, simultaneous collection of data on several related traits is highly desirable^21,22^.

In recent years, there has been much interest in high-throughput phenotyping (HTP), which allows the collection of data on multiple traits of interest at one time by using automated systems of data collection and analysis^21,22^. Most HTP protocols are based on low-resolution imaging using visible, multispectral, hyperspectral or fluorescence cameras, which allow the recording of traits both visible and invisible to the naked eye^23–31^. A major advantage of such data collection is a relatively short recording time and the possibility of storing and reanalyzing data at any time^25^. However, they might suffer from lack of specificity i.e. identification of the particular pest species.

Here, we present a semi-automated protocol to monitor *T. urticae* activity on *Arabidopsis thaliana* (L.) Heynh (*Brassicaceae*) plants by detection and analysis of leaf damage, eggs and black, digestive cells-derived feces. The central part of this protocol is an artificial intelligence (AI)-powered computer program, MITESPOTTER, which automatically analyzes high-resolution images of mite-infested *A. thaliana* leaves. The measurements revealed a wide variation in overall susceptibility/resistance among 14 *Arabidopsis* ecotypes as well as interesting patterns of symptom distribution among consecutive leaves of a rosette or between the upper and lower sides of a leaf, confirming the great potential of this protocol and AI in future research.

## Results

### Procedure description

To streamline the process of discovering the natural resistance of *A. thaliana* ecotypes to TSSM, we developed a semiautomated protocol that collects and measures unbiased data on the female oviposition rate, the area of damages and the area of fecal pellets. Our procedure consists of microscope scanning, producing high-resolution images of the leaf surface and image analysis with the specially developed AI-powered computer program MITESPOTTER. The protocol consists of manual and automated steps optimized to handle a task to screen approx. 1000 mite-infested *A. thaliana* plants within approximately 6 months engaging two skilled technicians.

**Figure 1:**
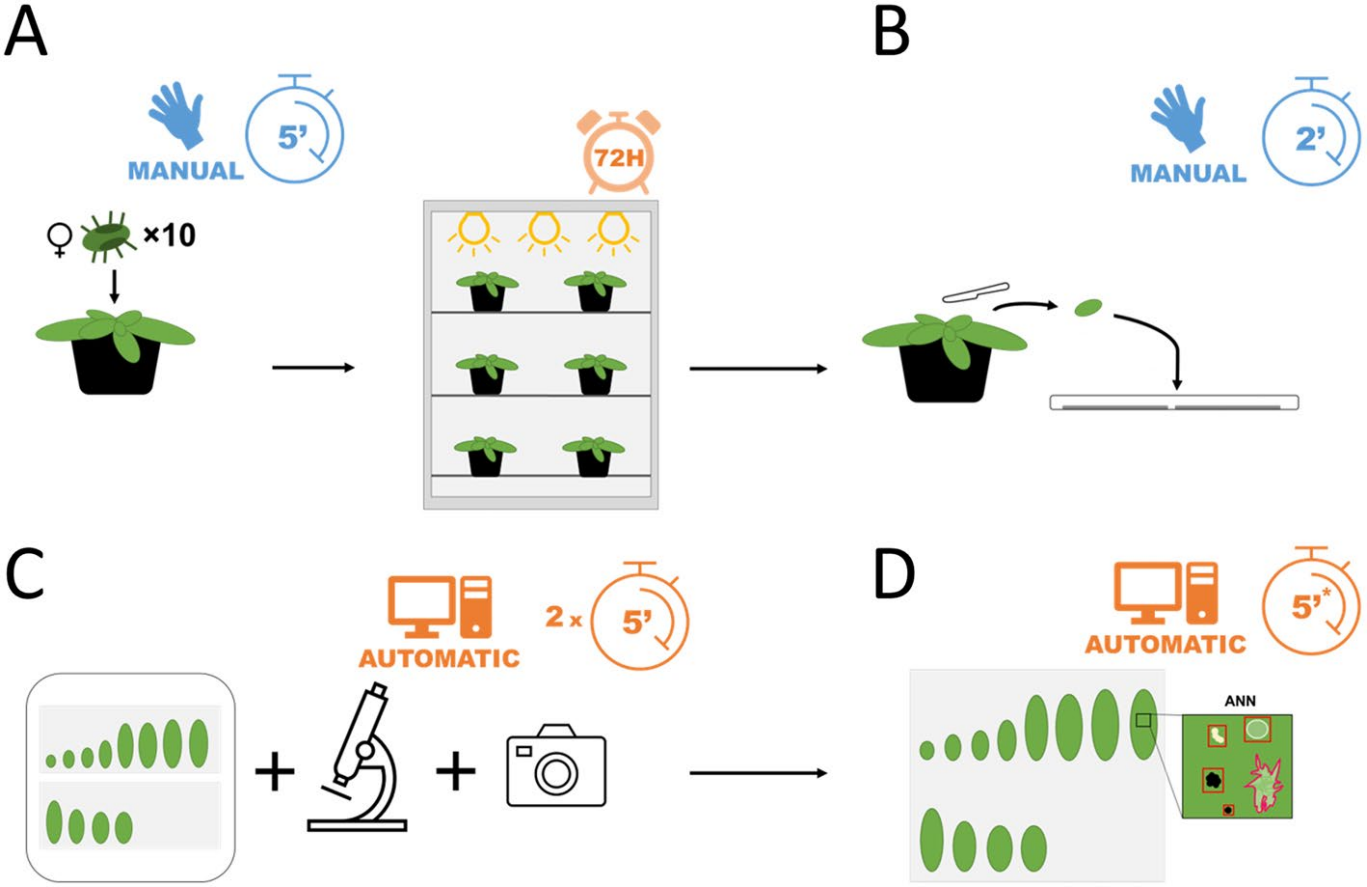
(**A**–**D**) Outline of the procedure with specified time needed for one plant during each step of the protocol with timer marked next to blue (manual) or orange (automatic) icon. The procedure was optimized for *A. thaliana* plants three weeks after seed stratification (4 °C) infested with 10 TSSM females for 72 h (**A**). There are three main stages of the procedure: preparing material for imaging (leaves are cut using a scalpel and placed immediately on tape on a square petri dish; (**B**), imaging (images should be recorded from both sides of the leaves); (**C**); image analysis (the MITESPOTTER program is used to measure the three main traits related to plants’ susceptibility to TSSM); and (**D**). *Analysis of a single image takes approximately 5 min., and analysis of 1000 images may take up to 10 h.

**Figure 2:**
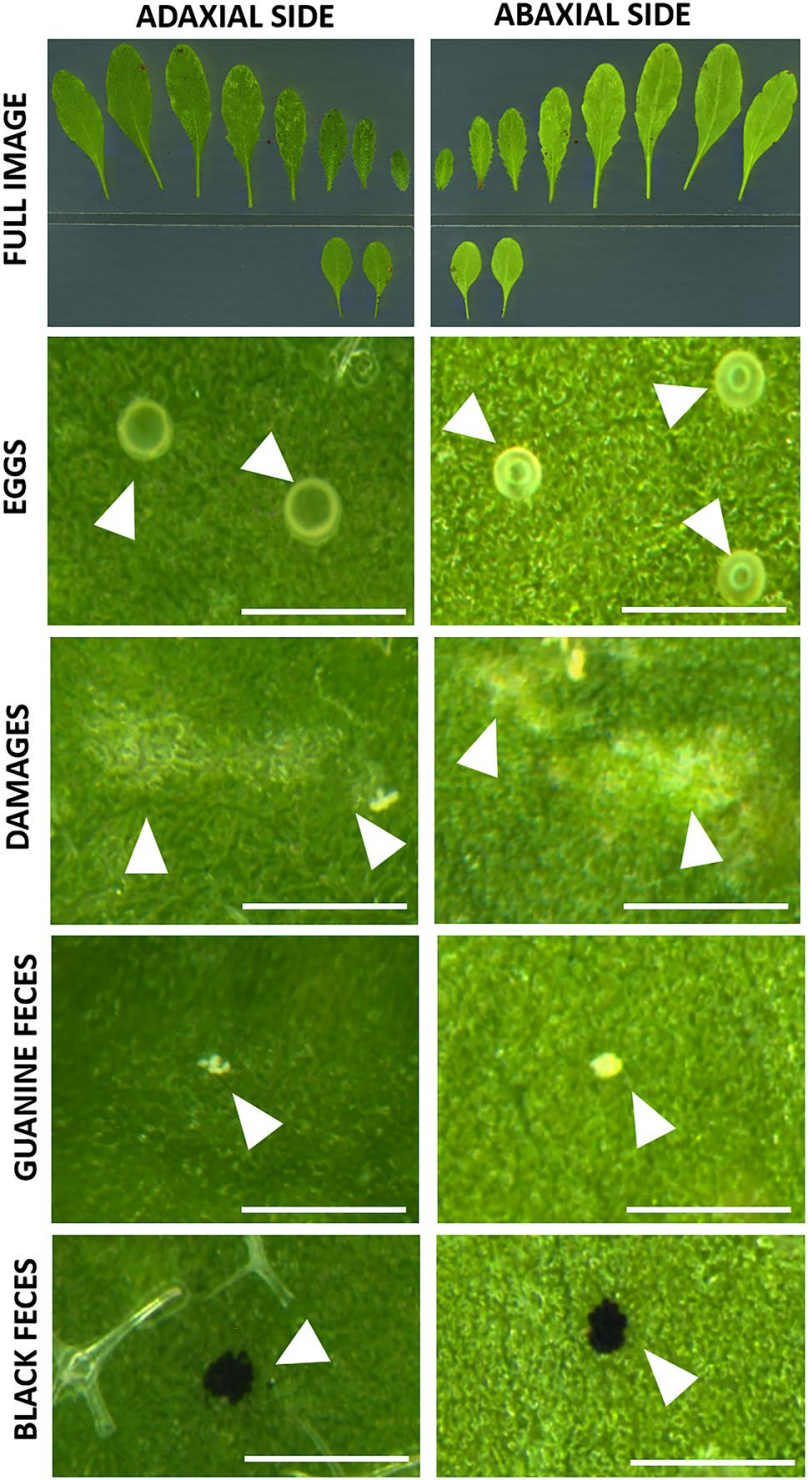
Images of abaxial (lower) and adaxial (upper) sides of *A. thaliana* leaves presenting traits (mite eggs, leaf damages, black, digestive cells-derived fecal pellets and white, guanine feces) related to the assessment of plant susceptibility to TSSM. Scale bar: 500 µm.

The procedure is outlined in Fig. 1 and presented in the 10 steps described below. Since the objects to be identified and measured should be recorded at a resolution sufficient to distinguish as many details as possible, we decided to use microscope scanning, giving a resolution of 8500 dpi with a pixel dimensions of approximately 3 µm. As a result, the initial image of max. 10 × 10 cm could be zoomed in to clearly distinguish the required details—eggs, irregular damages, black feces (Fig. 2) and many others. The high-resolution microscopic scans were performed with the same settings for the whole experiment and successfully subjected to automatic analysis with the specially developed computer program MITESPOTTER. The MITESPOTTER consists of the following modules: (1) image manager—cataloging full-size microscopic scans and decoding leaf side and accession, (2) leaf identifier—cataloging, numbering measuring surface and checking the abaxial/adaxial overlap of individual leaves, (3) object detection – segmenting leaf images, detection of eggs, damages and black feces by neural-network-based algorithms and marking them on unsegmented images, (4) visual presentation of results with optional manual adjustment, and (5) data export for downstream analyses.

### The main steps of the procedure

#### Preparing the material for imaging

**Figure 3:**
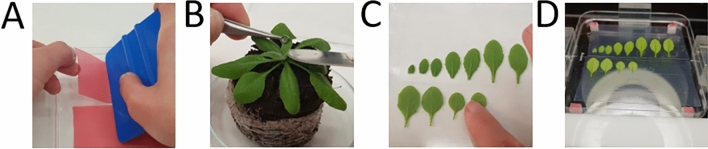
(**A–D**) The procedure of preparation of *A. thaliana* leaves for imaging consisted of four steps: (**A**) placing the double-sided transparent adhesive tape on the Petri dish lid; (**B**) cutting the plant’s leaves starting from the youngest using a scalpel blade, (**C**) sticking leaves with adaxial side facing the tape; (**D**) scanning plate on dedicated stand using the microscope and automatic table.

**Step 1.** Stick two 10 cm long (5 cm wide) pieces of double-sided transparent adhesive tape in parallel to the lid of a square 12 cm plastic Petri dish creating approx. 10 cm × 10 cm area for placing the leaves from one rosette (Fig. 3A). **IMPORTANT!** Use a plastic squeegee for better attachment of the adhesive tape and reduction of air bubbles because many of them may resemble mite eggs in obtained images of the adaxial side of the leaf.

**Step 2.** Cut off the leaves from the rosette starting from the youngest having a length of minimum 4 mm (smaller leaves are difficult to handle and are not subject to TSSM attack due to very dense trichome coating). Follow the natural phyllotaxy of a given rosette and end with the oldest (first leaf appearing above cotyledons; Fig. 3B). Place the leaves on the tape with the abaxial side up using bare fingers (latex or nitrile examination gloves and other hydrophobic intermediate layers for leaf contact caused detachment of numerous eggs from the abaxial leaf surface; Fig. 3C). **IMPORTANT!** Leaves should be prepared for scanning shortly before imaging to prevent the tape from misting up with humidity. It is crucial to keep a similar arrangement of leaves on the plates for each rosette: start from the left of the upper row and continue from the left of the lower row, leaving a few mm of clear spacing between leaves to allow automatic leaf detection and numbering during analysis.

#### Imaging

**Step 3.** Place the Petri dish with arranged leaves on the microscope stage with the leaf’s abaxial side up. Use a navigator (LasX program) to define the area that specifies the plate scan field, manually set the focus and start imaging by choosing the START button. When the scanning finishes, the merging process starts automatically (this option should be activated in the settings). Then, the Petri dish was turned over to record an image of the adaxial side of the leaf, and scanning was performed similarly. **IMPORTANT!** To minimize uncontrolled Petri dish displacements due to inertia during stage XY movements, a specific plate adaptor was made that additionally keeps the focus plane on the leaf when the Petri dish is turned over for imaging of the adaxial side of the leaf (Fig. 3D).

**Step 4.** Export the obtained image to a TIFF file by choosing the EXPORT IMAGE option in the LasX program menu. A new window will open where the file name and destination folder should be specified. **IMPORTANT!** The initial MITESPOTTER program configuration requires the file name to be created in such a way that it can identify the *A. thaliana* ecotype. The file name should contain information about: abaxial/adaxial side of leaf scanned, a given ecotype consecutive number and biological replicate, total number of leaves on the plate (e.g. D_A_1_I_2_K1_12). The obtained image can also be saved in the default LIF file format (Leica file), which can be opened with the LasX program and exported later to other graphical file formats. The imaging of one side of the plate produces files 3–4 GB in size; therefore, sufficient data storage capacity and PC performance should be ensured.

#### Image analysis

**Figure 4:**
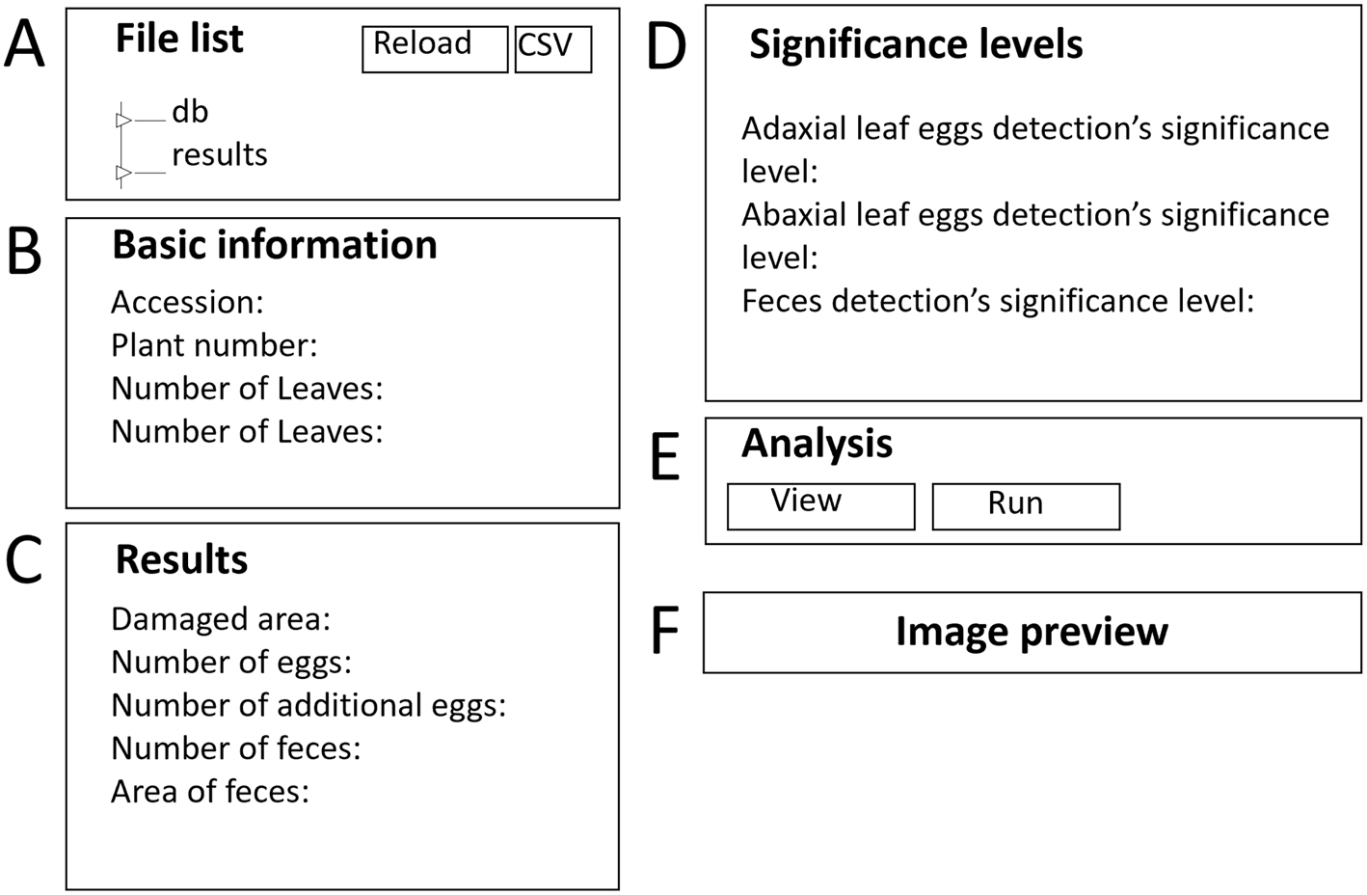
(**A–F**) Home page outline of the MITESPOTTER program. On the left side, the File list box (**A**) is presented with two folders: “db” and “results”. The “db” folder contains all the files that meet the criteria for analysis (TIFF format and proper file name). The “results” folder contains information for a single leaf. There are two buttons at the top of this box: RELOAD—to check if there are new files suitable for analysis, and CSV—to export data of all analyzed files to a CSV file. Next to the File list box, four boxes can be seen. The first two boxes, “Basic information” (**B**) and “Results” (**C**), present information (name of the ecotype), consecutive number of the plant analyzed, number of plant leaves, side of the leaves (abaxial/adaxial) and final results (of measured traits for whole plant of chosen image), respectively. The “Significance level” boxes (**D**) show the significance level used (default or manually selected) for the detection of eggs and black feces (the significance level for damage is fixed). The “Analysis box” (**E**) contains two buttons: VIEW (opens new window for analysis verification of chosen image) and RUN (visible only before analysis). The “Image preview box” (**F**) presents a miniature of a chosen image with all leaves of a given plant. Before the analysis, the boxes **B**, **C, D** and **F** on the home page are visible but without data or miniature images.

**Step 5.** Open the MITESPOTTER program and choose the RELOAD button in the File list box (Fig. 4A) to reload the file list (the file list is refreshed on demand or once a day due to image database size). **IMPORTANT!** The default automatic update of the file list is every day at 12 am.

**Step 6.** When the update is done (in our case, 1 h), choose the image for analysis and click the RUN button in the Analysis box (Fig. 4E) to perform the analysis of an unanalyzed pair of images of one plant (scans of the abaxial and adaxial sides of the leaf).

**Step 7.** After the analysis is performed, the information boxes on Fig. 4B,C,D,F will be filled with results and related data. Select the CSV button in the File list box (Fig. 4A) to export the data to a csv file. If verification of image analysis is needed, skip this step (go to step 8). **IMPORTANT!** The csv file contains columns with the name of the ecotype, the subsequent number of the plant (biological replicate), leaf side, and results for each side of a leaf separately, leaf area, area of damages, number of eggs, and number and area of black feces.

#### Visual presentation of results with optional manual adjustment

**Figure 5:**
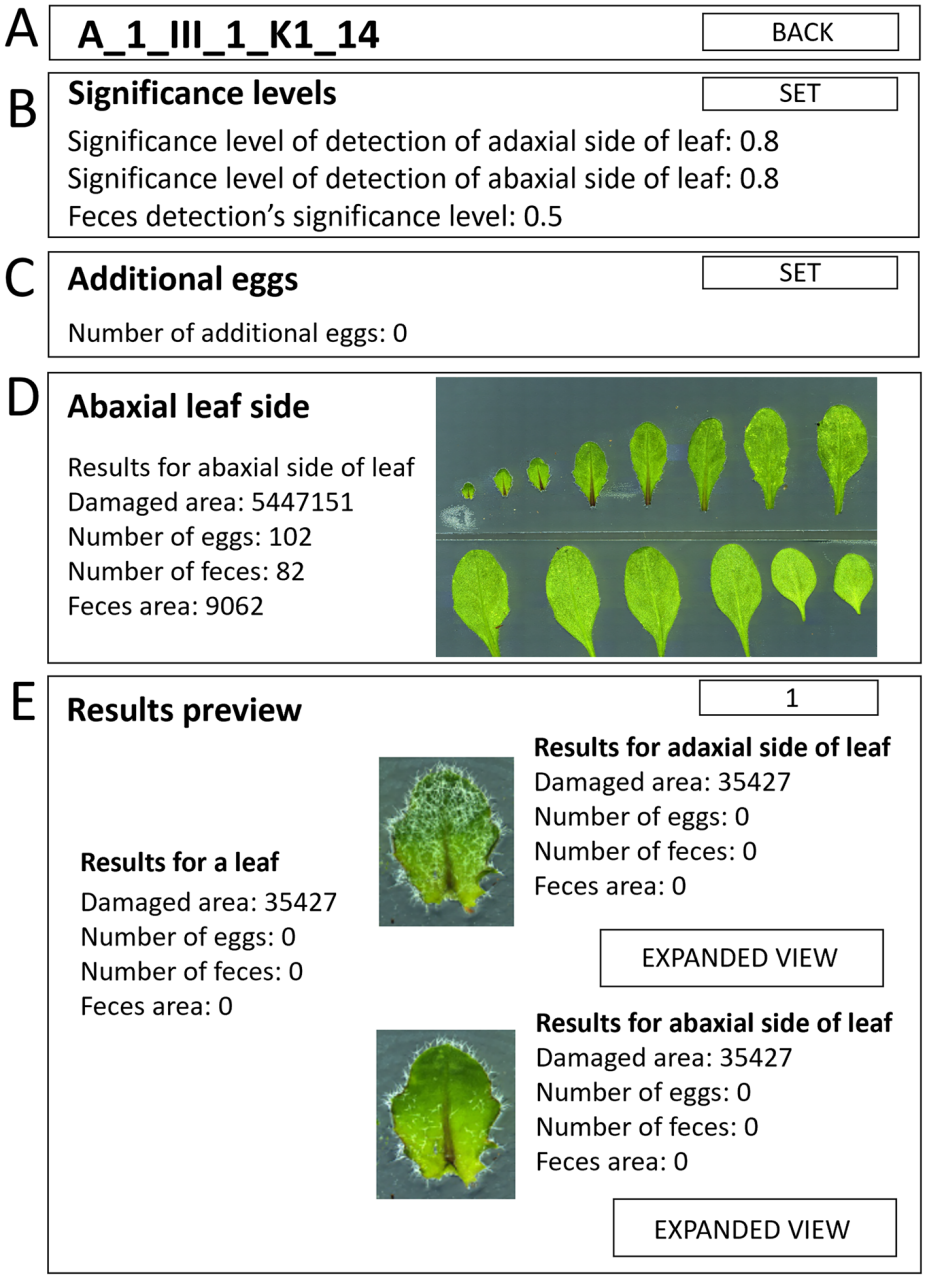
(**A–E**) MITESPOTTER individual plant window outline with detailed analysis, paired leaf images (adaxial and abaxial) and correction options after choosing the image for analysis. At the top, the chosen image’s file name and BACK button (going back to home page) can be seen (**A**). Below, four boxes can be seen. The “Significance level” box (**B**) shows and allows changes to the significance level of detection of adaxial/abaxial side of leaf currently used for detection of eggs and black feces. The “Additional eggs” box (**C**) presents and allows the addition of an extra number of eggs to the total number of eggs per plant (this is an option for eggs shed and seen outside the leaf, and they cannot be assigned to the specific leaf or leaf side; the eggs mistakenly identified or omitted on a leaf can be added or removed in the EXPANDED VIEW mode). The “Abaxial/Adaxial leaf side” box (**D**) contains the plant summary. The “Results preview” box (**E**) contains pairs of miniature images of both sides of an individual leaf. The pairs are lined up from the youngest to the oldest leaf with detailed results of the detection of traits for each leaf. The EXPANDED VIEW button allows seeing and the possibility of correcting the details of analysis on the full-resolution image.

**Figure 6:**
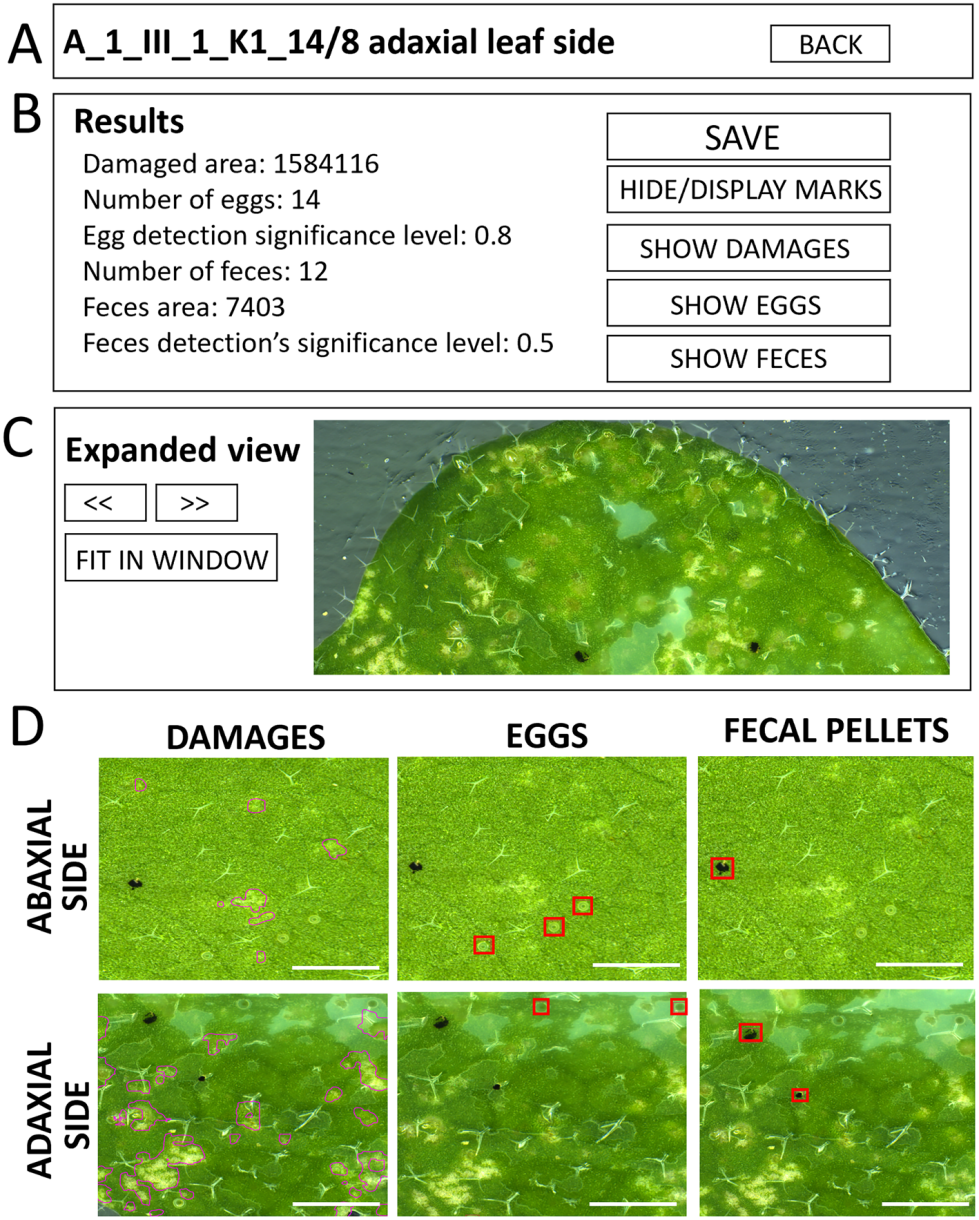
(**A–D**) MITESPOTTER “Expanded view” outline of a chosen leaf—(**A**) The “Results box” consists of two arrow buttons to navigate between successive leaves and FIT IN WINDOW button. The “Results box” (**B**) contains results and eggs and black feces detection significance level for the analyzed leaf image and five buttons: SAVE—to save changes made to detected objects (eggs and feces), HIDE/DISPLAY MARKS—to display or hide marks of detected trait (eggs/feces/damages), SHOW DAMAGES, SHOW EGGS and SHOW FECES—to see marked objects detected by MITESPOTTER or added manually (**C**) The “Expanded view box” presents an image with marked traits. Buttons: <  < and >  >—to change pictures, FIT IN WINDOW—resizes pictures to display size (**D**) exemplary traits (black feces, eggs, damages) marked in MITESPOTTER program. Scale bar: 1 mm.

**Step 8.** After the MITESPOTTER analysis is performed, a manual correction can be made. Select an image from the “File list” and press the VIEW button in the “Analysis box” (Fig. 4E). A new window will open (Fig. 5A–E). **IMPORTANT!** Visual inspection of MITESPOTTER results could be helpful when images were analyzed incorrectly in the following cases: extremely diverse leaf phenotype of a given ecotype or mutant, local loss of focus due to leaf curvature, significant egg shedding and dispersal outside the leaf area, significant misinterpretation of air bubbles as eggs or omission of eggs, the presence of chlorosis/necrosis not resulting from mite activity, etc. During such inspection, the significance threshold level can be changed, for black feces—within a range from 0.5 to 0.95, and for eggs—from 0.8 to 0.95 (Fig. 5B). The EXPANDED VIEW button next to a given leaf miniature icon (Fig. 5E) leads to the final high-resolution image of a leaf (adaxial or abaxial view) with detected objects visible on demand by pressing the buttons SHOW DAMAGES, SHOW EGGS or SHOW FECES (Fig. 6A–D).

**Step 9.** To remove misdiagnosed eggs or black feces from all detected ones, double click on the square marking an object and the program will ask if this is an object to be removed. To add unrecognized eggs draw a square with the mouse select tool. The changes should be confirmed by pressing the SAVE button (Fig. 6B). Use arrow buttons to navigate between high-resolution images of leaves and their sides of a given plant (Fig. 6C).

**Step. 10.** To export the data, go back using the BACK button in the right upper corner (Figs. 5A, 6A) to the start screen and follow step 7.

### Efficiency of MITESPOTTER analyses

MITESPOTTER efficiency was measured using two methods. The first method is an intrinsic part of the neural network model validation procedure. This procedure divides objects defined by experts into three groups with an 80-10-10 ratio, yielding an IOU (Intersection Over Union) value of 99.33% for correct pixel classification on the abaxial side of the leaf and 99.87% for the adaxial side. The second validation, conceptual, focused on entire identified objects rather than individual pixels. It entailed a comparison between evaluations conducted by MITESPOTTER and experts on six randomly selected leaves from various plants and ecotypes (black feces and damages), or six plants for egg count. This validation revealed an average efficiency of 101.63% for egg identification, ranging from 91.66% to 104.76% on individual plants; 100.73% for black feces area, ranging from 100.55% to 100.97% on individual leaves; and 102.70% for damages, ranging from 95.57% to 106.77% on individual leaves (see Supplementary Fig. S1 for details). Statistical analysis indicated no significant difference between the evaluations conducted by MITESPOTTER and the experts (p ≤ 0.05).

### *A. thaliana* susceptibility to TSSM measured by MITESPOTTER

In this study, the susceptibility of 14 *A. thaliana* ecotypes to TSSM was determined based on three traits—the area of damages on leaves, female oviposition rate and black feces area measured by the MITESPOTTER program. With this program, we could have avoided splitting the large number of experimental plants into groups to be analyzed at one time. However, such a scenario (splitting a large tested population) is still realistic if over 1000 plants are to be analyzed. Then, seasonal changes may influence the results, even though the initial rearing of the *T. urticeae* population, age-synchronization of females and infestation were performed in stable temperature, light and humidity. Therefore, the presented measurements were made in three repetitions at six-week intervals, and a reference ecotype Col-0 was added to every group of plants tested.

**Figure 7:**
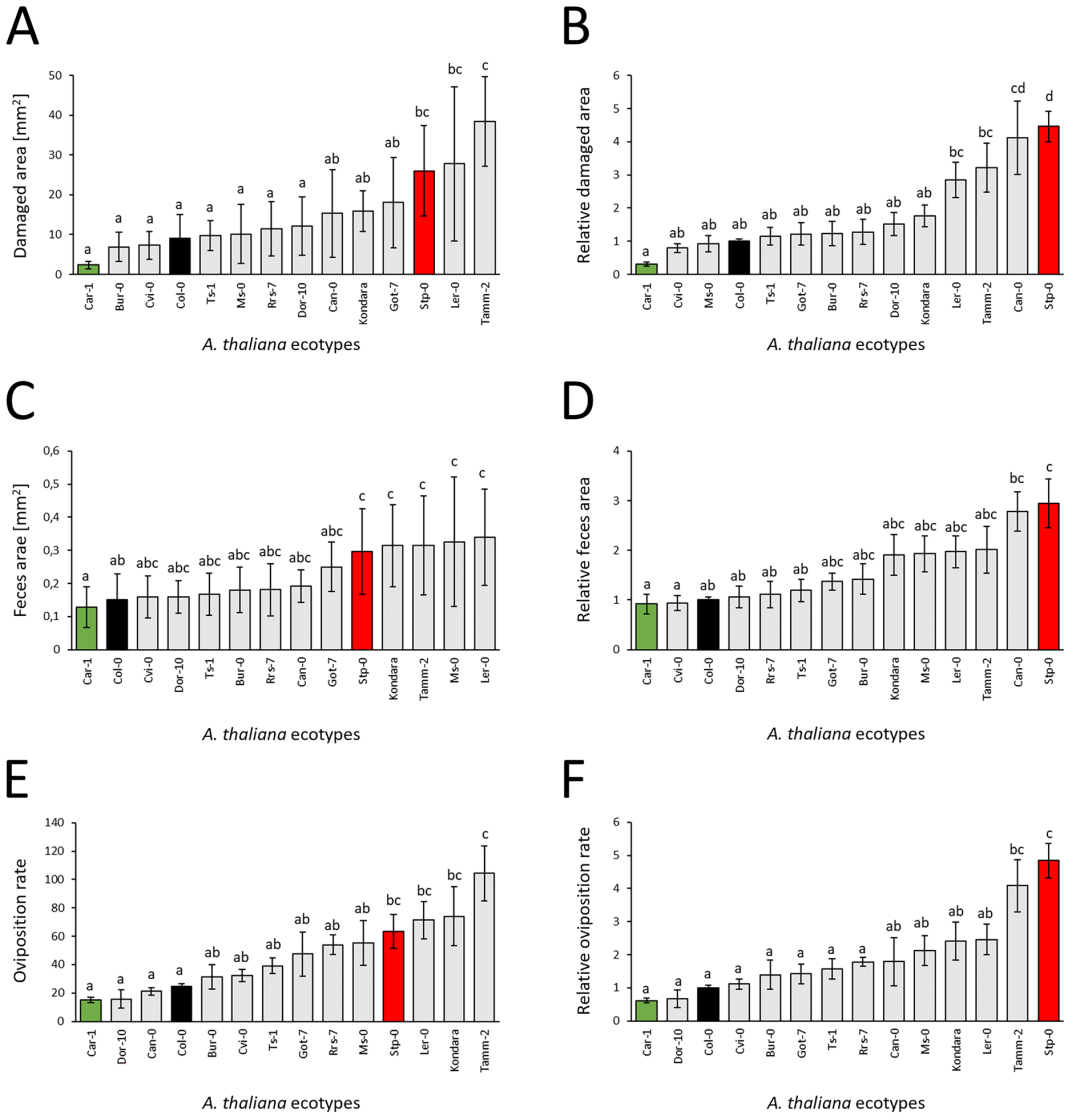
(**A**–**F**) Susceptibility of 14 *A. thaliana* ecotypes to TSSM measured by the area of damages (**A**,**B**), oviposition rate (**C**,**D**), and black feces area (**E**,**F**) after 72 h of infestation with 10 TSSM adult females per plant. The results present the distribution of absolute (**A**,**C**,**E**) and relative (**B**,**D**,**F**) values among the 14 ecotypes. The least susceptible ecotype, Car-1, is marked in green, the most susceptible ecotype, Stp-0, is marked in red, and the reference ecotype, Col-0, is marked in black. The letters a, b, c, d and their combinations denote statistically significant (p ≤ 0.05) differences between ecotypes for a given trait using the Compact Letter Display method of the ANOVA test. Means ± SE are shown based on the analysis of six independent biological replicates.

The results are presented as absolute and relative values. Absolute values are given for feeding symptoms and black feces area, measured in mm2, as well as oviposition rate, represented as the number of eggs per female per plant. Relative values were calculated by dividing the absolute results by the mean reference ecotype values, derived from 6 plants for each repetition (Fig. 7A–F). Among the examined ecotypes, the least susceptible was Car-1 irrespective of whether absolute or relative values were considered in each of three susceptibility determinants. When the absolute and relative trait values were considered, the most susceptible ecotypes were Tamm-2 and Stp-0, respectively (Fig. 7A–D). This was true for the area of damages and oviposition rate, whereas in the case of black feces area, the comparison of absolute values failed to indicate with statistical significance a single most susceptible ecotype (Fig. 7E,F). The detailed lineup of the tested accessions also varied slightly depending on whether absolute or relative values were considered and the susceptibility determinants used. In summary, susceptibility based on the criterion of relative area of damages varied more than 18-fold between outermost *A. thaliana* ecotypes. However, for the oviposition rate, the dynamic range was approximately eightfold, whereas for the black feces area, it was more than threefold (Table S2).

### Distribution of TSSM symptoms on *A. thaliana* leaves

**Figure 8:**
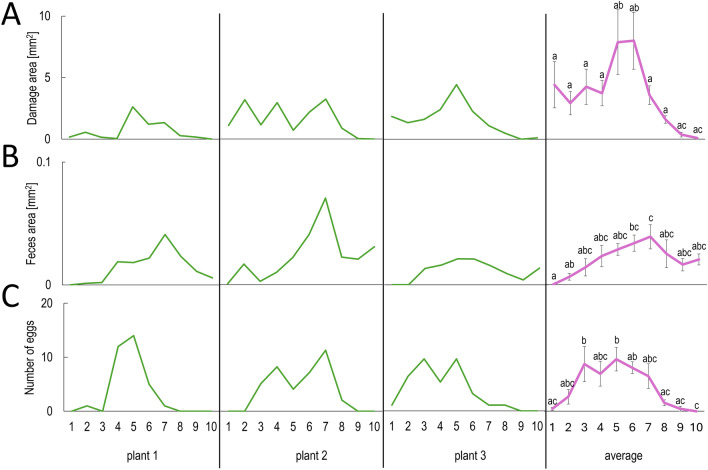
(**A–C**) A comparison of trait distribution (**A**—area of damages, **B**—black feces area, **C**—number of eggs laid,) on consecutive leaves of the rosette (starting from the oldest) of *A. thaliana* Col-0 ecotype (3 randomly selected individual plants and an average value for 6 plants). The letters a, b, c, and their combinations denote statistically significant (p ≤ 0.05) differences between leaves for a given trait using the Compact Letter Display method of the ANOVA test. Means ± SE are shown based on the analysis of six independent biological replicates.

**Figure 9:**
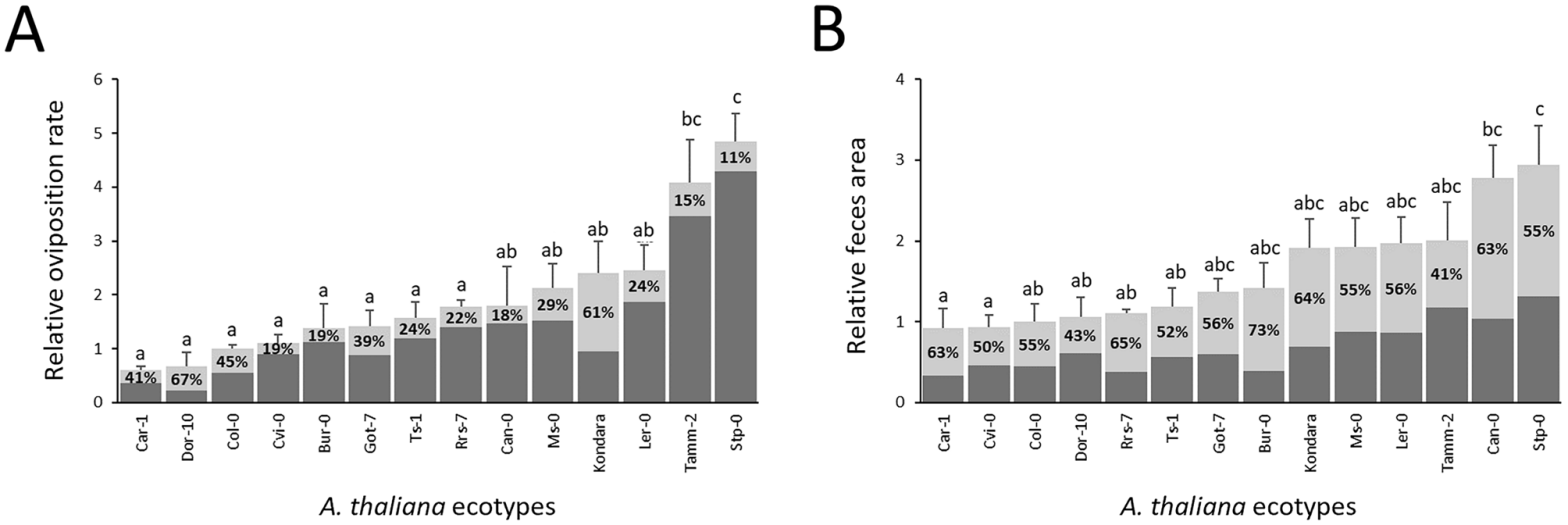
(**A,B**) Distribution of eggs (**A**) and black feces (**B**) on the abaxial (dark gray) and adaxial (light gray and %) sides of leaves expressed as the relative measure of a given trait. The letters a, b, c, and their combinations denote statistically significant (p ≤ 0.05) differences between ecotypes using the Compact Letter Display method of the ANOVA test. Means ± SE are shown based on the analysis of six independent biological replicates.

The experimental design used for this study and the MITESPOTTER computer program allow an examination of the *A. thaliana* ecotype’s susceptibility to TSSM based on the distribution of leaf damages, female oviposition rate and black feces area on consecutive rosette leaves (Fig. 8) as well as showing the proportion of eggs and feces on abaxial and adaxial leaf surfaces (Fig. 9). Analysis of the distribution of these traits on consecutive leaves sometimes showed repetitive highly frequent symptoms on some and very little incidence on others (Fig. 8). Such a pattern was not conserved for all individual plants of the same ecotype but was visible for approximately 50% of plants where repetitive peaks of mite activity were observed (Fig. S1).

For all tested plants and ecotypes, there were no eggs and damages on the youngest leaves, which may be a result of dense trichomes forming a physical barrier for females. The mature, fully grown and expanded leaves did not form trichomes de novo*,* and their density even on the adaxial side of the leaf did not interfere with female activity. Despite the rather random distribution of symptoms on consecutive leaves, the average values suggest a slight preference for a behavioral sequence during mite migration across the rosette, moving from the youngest leaves to the oldest: excretion, feeding, and egg laying (Fig. 8). The analysis of such data presents intriguing perspectives for studies on plant-mite interactions.

The ratio of eggs on the adaxial/abaxial side of leaves was mainly approximately 1:5, which corresponds with TSSM’s nature of staying mostly on the abaxial side of the leaf. However, in the case of two ecotypes (Dor-10 and Ms-0), approximately 60% of eggs were laid on the adaxial side, and in the case of two other ecotypes (Col-0 and Bur-0), approximately 40% of eggs were found on the adaxial side (Fig. 9A). The percentage of black feces detected on the adaxial side ranged from 41 to 73% (Fig. 9B).

## Discussion

### Approaches to monitoring plant-spider mite interactions

Studying the interactions between herbivorous pests and host plants may be a starting point of research focusing on the herbivore side (e.g., adaptation) or the host plant (e.g., defense mechanisms). Such research often requires a tedious inspection and classification of pest population structure and dynamics as well as the damage symptoms on hundreds of plants, creating a bottleneck in the availability of qualified experts. In particular, sustainable agriculture in the era of climate warming requires an acceleration of resistance breeding based on well-described molecular mechanisms, which is difficult to accomplish without precise and reproducible assessment of genetically and phenotypically polymorphic populations of a given plant species, allowing individual classification as susceptible or resistant^32^.

Plant resistance-related traits can be defined on the basis of pest performance characteristics, for example, the number of individuals (or their DNA content), population structure, oviposition rate, and developmental time^15^, or by the quantification of their effects on host plants, e.g., chlorophyll loss^33^, marker gene expression, content of plant secondary metabolites^25,34^, biomass loss, proportion of tissue lesions or necrosis and other feeding symptoms^35,36^.

In the case of plant-spider mite interactions, modern methods to assess infestation are often based on the analysis of visible or multispectral images recorded by low-resolution cameras and subsequent calculation of vegetation indices (e.g.^27,31^). Such methods allow correlation of observed symptoms to spider mite infestation and can sometimes distinguish the effects of the activity of two different pest species and plant malnutrition symptoms^26^. Such methods are ideal for the monitoring of a given species on a given plant variety for the optimization of crop protection,however, they still lack robustness in terms of natural variability of pest races and developmental stages or host genotype (cultivars, breeding lines, segregating progenies, ecotypes, mutants. New developments in neural network models provide some hope for improving this, especially for images recorded in variable light conditions^23^. Obviously, these methods still lack the versatility of a human expert and are rather limited to a particular task and budget. An interesting and smart tradeoff of cost, throughput and expert engagement without sophisticated equipment was shown by Cazaux et al.^37^, who developed a method to assess the susceptibility of *A. thaliana* to TSSM by measuring damage by a semiautomated selection of the altered area on a fine square grid over leaf images generated by an office scanner. The protocol provided here fully automatizes this process, requiring only 1,000 examples of the trait’s images for neural network training.

### Optimizing microscopic scanning and image analysis

The presented protocol, which includes microscope scanning of TSSM-infested leaves and AI-aided image analysis using the MITESPOTTER program, allows large amounts of data to be obtained regarding plant resistance to TSSM. It accelerates the assessment, standardizes the criteria, measures precisely, and reduces the engagement time of experienced specialists. Moreover, the data can be stored and reanalyzed, and the adaptation of the protocol to other pest-host plant models is not complicated.

During the optimization of the protocol, several problems were solved. For example, the recorded leaf damage, eggs and fecal pellets differed between adaxial and abaxial scans. This was because the abaxial side was photographed directly, whereas the imaging of the adaxial side passed through the plastic plate and adhesive tape, causing different light scattering. Moreover, the adhesive tape added noise disturbing the detection of eggs due to small air bubbles present in the tape glue, which sometimes could be mistaken for eggs. Some additional noise may also originate from leaves stuck unevenly to the tape forming closed spaces, which could quickly steam up by transpiration, disturbing subsequent imaging and detection.

Consequently, for separate neural network training, abaxial and adaxial training sets were prepared for eggs and leaf damage. In the case of eggs, another image-diversifying factor was the egg age, which could vary from 0 to 3 days. Therefore, the training set should contain transparent, straw yellow, and orange eggs. In the case of leaf damages, their shape and area also varied depending on the side of the leaf and overlapped only partially. This could depend on which mesophyll cells were damaged (sponge, palisade or both). Therefore, in the MITESPOTTER configuration, we decided to conduct damage detection of the two sides separately. Then, the area was summarized and plotted on both sides in the same shape (the overlapping portion was not duplicated). Another problem concerning mainly the adaxial side of the leaves was caused by trichomes, which often obscured the objects to be detected. Admittedly, the trichomes interfered with object detection on the youngest leaves with the highest trichome density. Fortunately, trichome density was also an efficient physical barrier causing these leaves to be unwillingly chosen by mites to feed on and lay eggs. On the older, more expanded leaves the trichomes were no longer a barrier for mites, as well as the chosen neural network model efficiently ignored the trichomes.

In the proposed protocol, we have also incorporated a rarely-used parameter: the quantity of black feces^1^. The fecal pellets of the TSSM comprise digestive cells in various stages, predominantly black when observed under visible light. Additionally, the TSSM excretes nitrogenous waste in the form of discrete cream-colored guanine spherules (white feces,Fig. 2), alongside the light inclusions into the black digestive cells^38^. However, the detection of guanine feces on leaves proved challenging, both during the training set preparation and neural network object detection. Consequently, we opted to analyze only black feces in this study.

**Figure 10:**
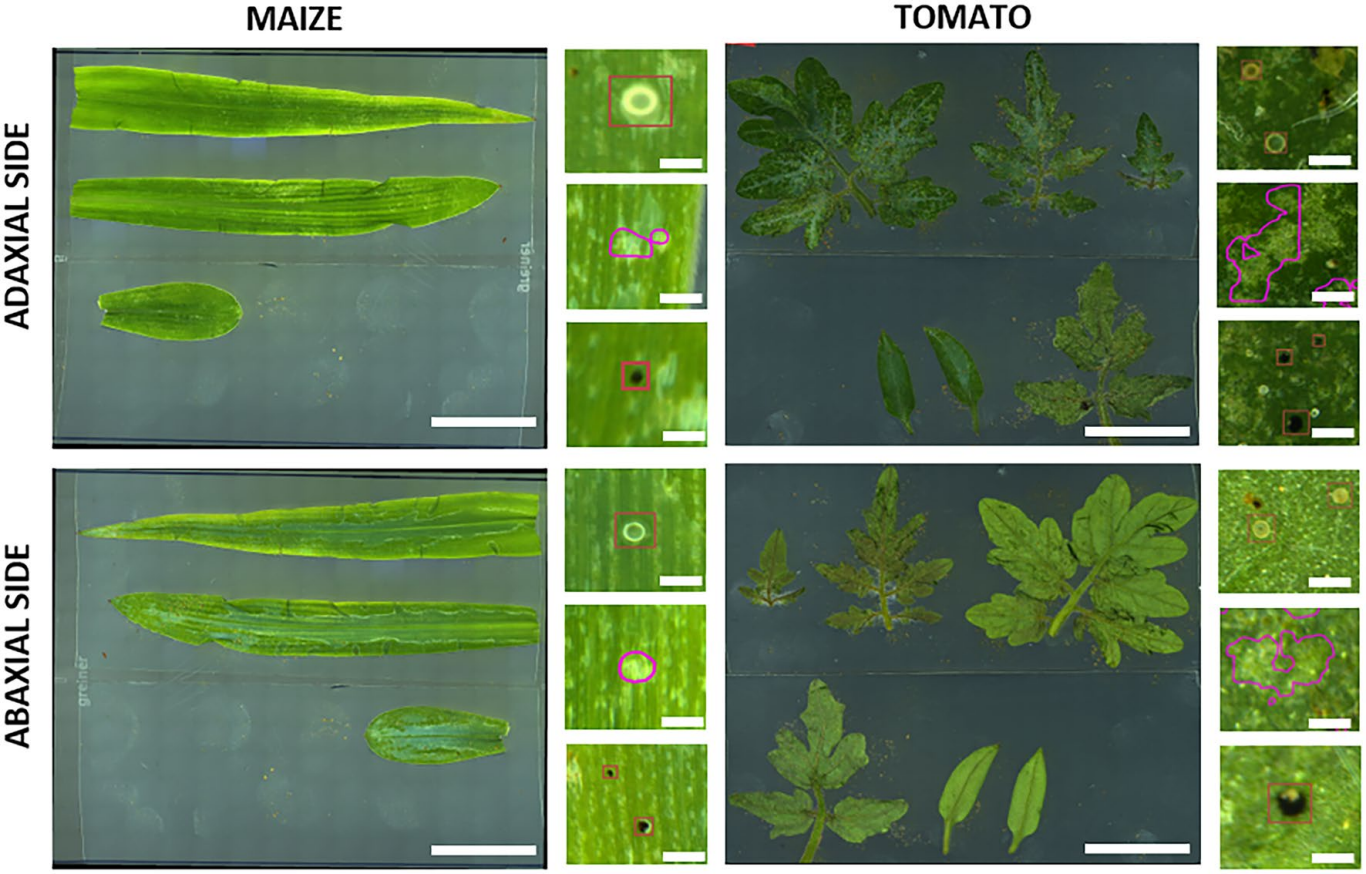
Scans of leaves from plant species other than *A. thaliana*. The current procedure adapted for *A. thaliana* was applied to maize seedlings (10 days post germination) and tomato plants (21 days post germination). While the procedure is generally effective, optimization of neural network models is necessary. Scale bars: 2 cm - full scan, 200 µm - symptoms closeups.

All the optimization points mentioned above necessitate a detailed review of the initial results to facilitate final decisions on the default settings of the MITESPOTTER program. Therefore, the program includes a module for comprehensive visual presentation of results, with the option for manual adjustments (see Fig. 6). This module proves particularly valuable when incorporating new species or traits into the program. It plays a crucial role in providing guidance for optimizing neural networks, as demonstrated in the case of tomato and maize (see Fig. 10).

### Improving the procedure throughput

The presented protocol allows for the assessment of the activity of TSSM on hundreds of plants. There is the potential to optimize it for other plant-pest interactions (Fig. 10). We are also aware that several bottlenecks exist, and further optimization would increase the protocol’s performance. This includes its applicability to analyze samples collected in field experiments or those infested/infected with multiple pests or pathogens. Below are a few issues where we see a realistic chance for improvements.

1. Infestation—in our protocol, we arduously applied 10 young females from a synchronized population per plant, but there are a few alternative options (the trade-off is that they give a new source of variability within the experiment):
  Massive infestation without synchronization or selection—all stages of larvae and adults are transferred to plants, e.g., by brush (for a smaller number of mites per plant) or by pump for more than 20 females per plant^37,39^. Using this type of infestation, the measured resistance/susceptibility may be more variable because (a) not every female will be at the peak of its fecundity, (b) larvae, nymphs and males do not lay eggs and (c) the structure of the population used may vary.
  Free migration—“free choice” scenario—plants are put into the stock colony of TSSM (where mites of all stages of their life cycle are present) or overpopulated leaves from the stock colony are placed on top of experimental plants. Mites have the freedom to migrate, which depends on the maternal population host species/ecotype and the colony density, as well as the physical proximity/contact of leaves. After the experiment, the symptoms can be measured as in the presented protocol, or the number of mites can be assessed by brushing out the mites to double-sided adhesive tape and scanning it using a similar imaging system as above.
2. Fewer leaves to be analyzed—in the case of *A. thaliana,* plants a few days younger could allow all leaves to fit in one row, reducing the time required for specimen preparation and scanning. Different growth intensities of *A. thaliana* ecotypes should be considered as well as the possible susceptibility discrepancy between younger and older seedlings. Such optimization would not be possible with other species having larger leaf sizes and numbers where specific sampling protocols should be elaborated to provide statistically significant results.
3. Image quality—the full 10 × 10 cm area scan is composed of approx. 400 merged camera shots. A higher resolution of the camera and a larger field of view of the microscope optics would allow for a reduction in the number of pictures taken from each specimen.
4. Image size—the uncompressed image exceeds 3 GB, implying quite high requirements with respect to storage capacity and computer performance for downstream analyses. Considering progress in computer hardware engineering, we expect gradual improvement—reduction of computing time.

### Example study—trait variability among *A. thaliana* ecotypes

Trait variability among the 14 ecotypes tested here showed dynamic ranges of 18.4, 7.9 and 3.2 for the relative values of the area of damages, oviposition rate and black feces area, respectively. The values obtained suggest polygenic determination of these traits similar to the results obtained by Zhurov et al.^39^, where susceptibility was assessed by measurement of the damaged leaf area. In addition to a different method of measurements in our experiments and the cited report (MITESPOTTER vs. manual area selection, three days after infestation vs. four; three- to five-day-old unadapted and synchronized females vs. unadapted females manually selected from unsynchronized population), the most and least susceptible *A. thaliana* accessions show similar dynamic ranges of the damaged area.

The three characteristics presented here result from very complex biological processes, suggesting that a diverse genetic repertoire may be responsible for its measured value^1^. Moreover, we used synchronized but unadapted females. Despite these factors, the area of damages, female fecundity and black feces area indicate the same ecotypes as the most and least susceptible (the detailed rank of other ecotypes differ slightly depending on the trait measured), indicating the existence of a general antixenosis/antibiosis mechanism in the *T. urticae*–*A. thaliana* interaction. Deciphering the molecular background of this variation is of the highest importance for both basic research and plant breeding. Another highly desired follow-up research would be addressing the question to what extent the presented ecotype variation would be reduced if the host adaptation step is used^40–42^.

### Example study—distribution of TSSM symptoms on leaves

The described method allows measurement of the symptoms on each leaf individually, sometimes showing peaks and valleys on the chart (Fig. 8). Under natural conditions, the mite population tends to colonize plants with an aggregated distribution pattern. This may be due to unevenly spread resources, varying environments, less species-specific competition and negative antagonism^43^. In our experiments, these factors are rather negligible due to the low density of synchronized females as the only herbivore on the tested plants, however, we see great potential for using our method in research on mite population dispersion within and between plants. On the other hand, the observed patterns of symptom distribution within one plant may be associated with the parastichy-dependent distribution of systemic acquired resistance (SAR) signals^44–46^. Parastichy in *Arabidopsis* describes direct or indirect vascular connections of leaves formed during development and depends on the orientation of phyllotaxy and the rosette size and can follow the n + 3, n + 5 or n + 8 pattern^47^. The best results in the cited literature, however, were obtained when only one leaf was infected/infested and SAR distribution was monitored by marker gene expression. In the case of our experiments, mite migration and activity did not produce convincing parastichy patterns, suggesting that rosette topology (mainly the shoot apex where young females were applied) and random leaf contact within the rosette caused unequal distribution of females and symptoms. This does not exclude the SAR influence on representatives of the same or different species^48^, but the experimental setup applied made it impossible to observe it. The individual measurements of leaf symptoms by the MITESOPTTER program may also be useful in other experiments where, e.g., plant canopy distribution of pests or pathogens is important.

An interesting part of our results was also the observation that there is variability among ecotypes in the distribution of traits depending on the leaf side (number of eggs and black feces area; the damaged area detected largely overlaps on the abaxial and adaxial side). Several factors may contribute to such variations, typically linked to the host plant species, environment, or the community of species inhabiting the same plant. Assuming no variability in host species or environment (in a controlled setting such as a growth chamber), the variation observed can be attributed to the innate physical, chemical, or molecular factors of the host plant ecotypes analyzed. This presents intriguing area for future exploration.

### Downstream applications of collected data

Forward genetics, i.e. linking a specific phenotypic trait to single or multiple genes is crucial for plant breeding and basic research. The most precise approaches require screening of a trait variation in extensively polymorphic and large populations. The advent of relatively low-cost next-generation sequencing methods has provided an incredible source of genetic polymorphism data; however, phenotypic trait assessment and quantification are still difficult to simplify and accelerate. The presented protocol is designed to collect data on phenotype variation with a specific focus on traits related to TSSM susceptibility of *A. thaliana* ecotypes where hundreds of them are fully sequenced. Such data can be used in genome-wide association study (GWAS) or transcriptome-wide association study (TWAS) to identify plant genes or genetic markers to be used in TSSM resistance breeding^49,50^. Such experiments, however, would require screening of mite susceptibility of many more than 14 ecotypes or segregating progeny (100–200)^51^.

## Conclusion

We have developed an innovative AI-powered computer program called MITESPOTTER, known for its exceptional accuracy in detecting and measuring TSSM feeding symptoms. This advanced tool efficiently and precisely assessed the area of leaf damages, quantified the number of eggs, and determined the area of black feces across all rosette leaves of 14 *A. thaliana* ecotypes simultaneously. We implemented this program in the plant susceptibility/resistance assessment protocol based on high-resolution microscope imaging of both leaf surfaces infested by TSSM. This protocol can be adapted to other small pest-host plant interactions. The initial results show great potential for our method in studying the distribution of mite pests both among potential host plants and/or within the plant canopy. Specifically, among the tested *A. thaliana* ecotypes, we observed that Stp-0 exhibited the highest susceptibility to TSSM, while Car-1 demonstrated the lowest susceptibility. Fecal distribution on both leaf surfaces appeared uniform across all tested ecotypes, with a slight prevalence observed on the adaxial side. Conversely, eggs were predominantly laid on the abaxial side of the leaf in most ecotypes, except for the Ms-0 ecotype, where the opposite pattern was observed. The protocol presented here is prepared for screening large, genetically diverse populations of *A. thaliana* infested with *T. urticae*, facilitating the rapid identification of genes contributing to enhanced pest resistance.

## Materials and methods

### TSSM age‑synchronized colony

A TSSM age‑synchronized population was obtained according to Barczak-Brzyżek et al.^52^ with some modifications. To synchronize the age of females, freshly detached leaves of three-week-old plants of the common bean (*Phaseolus vulgaris* L. cv. Ferrari, PNOS, Ożarów Mazowiecki, Poland) were placed on wet cotton in Petri dishes (150 mm in diameter) with the abaxial (lower) side up, and ten young females from the stock lab colony maintained on common bean plants (for more than 150 generations) were transferred to each of 10 detached leaves. The Petri dishes were placed in a growth chamber (SANYO Plant Growth Chambers MLR-350H) under controlled conditions (photoperiod—16/8 h day/night; light intensity: 150 µmol photons m^−2^ s^−1^, relative humidity, RH—65 ± 10% and temp.—23 ± 1 °C) to let the females lay eggs for 24 h. Then, the females were removed, and when the larvae hatched, they were moved to two-week-old common bean plants growing in the growth chamber to allow the females to develop synchronously.

### TSSM infestation of *A. thaliana* ecotypes

Fourteen *A. thaliana* L. accessions, originally derived from ecotypes found and collected in various world locations and referred to in this paper as ecotypes, were acquired from the Nottingham Arabidopsis Stock Centre (NASC; Nottingham, UK): Car-1 (NASC id: 76742), Cvi-0 (76789), Ms-0 (76555), Col-0 (76778), Ts-1 (76615), Got-7 (76495), Bur-0 (N6643), Rrs-7 (76593), Dor-10 (76806), Kondara (76532), Ler-0 (77020), Tamm-2 (76610), Can-0 (38909), and Stp-0 (77283) (Table S1). NASC routinely provides seed and information resources to the research community. Ordering and working with *A. thaliana* ecotypes from NASC were done in compliance with European and institutional guidelines. The plants were sown on peat pellets (Jiffy, Netherlands), stratified for two days at 4 °C and grown for three weeks under controlled conditions (16/8 h (day/night) photoperiod; 150 µmol photons m^−2^ s^−1^ light intensity, 70% RH and temp. 22 °C).

Ten five-day-old female mites originating from the age-synchronized colony were transferred using a fine brush (diameter 1.8 mm) to the youngest leaves of the rosette of each *A. thaliana* experimental plant. Females had free choice to feed and lay eggs on each rosette leaf for 72 h. Mite-infested *A. thaliana* plants were placed in the growth chamber under the same controlled conditions as described above.

### Imaging of mite-infested *A. thaliana* leaves

All *A. thaliana* rosette leaves from a single mite-infested plant were cut using a scalpel and arranged on a square Petri dish lid (120 × 120 mm) covered with double-sided transparent adhesive tape starting from the youngest (4 mm long leaf) to the oldest leaf excluding cotyledons.

The imaging of leaves differing in size and shape was performed using a specially configured Leica Thunder Imager System (Leica Microsystems Ltd., Switzerland) consisting of an M205 FA stereo microscope with a DFC7000 T camera and fully motorized XY-Scanning Stage (Märzhäuser Wetzlar GmbH & Co. KG, Germany). The acquisition software Leica Application Suite X (LasX; Leica Microsystems CMS GmbH, Germany) was operated on a PC (HP Z4 G4 Workstation, Hewlett-Packard Company, Palo Alto CA, USA with a NIVIDA GeForce RTX 2080 Ti graphics card, Nvidia Corporation, Santa Clara CA, USA). Image acquisition was performed using reflected light (LED5000 High Diffuse Dome Illuminator; Leica Microsystems Ltd., Switzerland) under 25 × magnification. Using a navigator tool from LasX software, the image acquisition area and focus were set manually. A full leaf scan included approximately four hundred images of neighboring and partially overlapping pictures, which were merged automatically after scanning with the imaging system software. The images of abaxially and adaxially scanned leaf surfaces were exported to TIFF format and stored on a QNAP TS431 disc array (QNAP Systems Inc., New Taipei City, Taiwan).

### Image analysis

The analysis of high-resolution images of TSSM-infested *A. thaliana* leaves was performed by a computer program, MITESPOTTER (developed specifically for this project), detecting and determining the number of eggs laid by females, the area of feeding damage, and the amount (number and area) of fecal pellets. All graphical data management and specific analyses, including image cataloging, leaf measurement and indexing, object detection and quantification, were performed using MITESPOTTER. The program includes optional manual correction of the results and data export for downstream analyses. The MITESPOTTER is a web-based application written in Python programming language, utilizing the Django library. The program takes whole plant scans as input and autonomously executes various processes, such as image segmentation, neural network activation, object detection, and calculation of network confidence thresholds. The results are exported in CSV format.

### Neural networks

The detection of eggs, leaf damage, and fecal pellets by the MITESPOTTER program was based on neural networks and required prior network training with manually marked items.

The identification of eggs and black feces was treated as an object detection problem. In deep learning, object detection finds instances within an image that fulfil the criteria of the given neural network. The bounding box that surrounds the items found is rectangular. Detected objects are counted and described by position, dimensions, and area. These tasks were performed using the Detectron2 library created by Facebook AI Research^53^.

To detect black feces, we used a common architecture in object detection problems—Faster RCNN. The Faster RCNN is based on a two-stage algorithm. These types of models are built from two algorithms. The first of them finds candidate objects in the image. The second stage is the verification of every candidate and generating features such as a bounding box for each candidate. In the Faster RCNN^54^, in the first stage, we used Resnet50^55^ to generate the proposal regions. The rest of the network architecture is standard for this model. The eggs were detected similarly using the Faster RCNN approach.

The identification of feeding damage was treated as a semantic segmentation task. Image segmentation is the task of classifying each pixel. This means assigning each pixel to one category from a defining set. Every pixel on our image may or may not be classified as a symptom area. This allows the determination of the size, shape, and area of the objects of interest. This problem was solved by a feature pyramid network (FPN)^56^. This network represents the encoder-decoder type of architecture. In these models, the encoder network generates an abstract representation of an image. In the second step, the decoder network from this representation creates a fine output. As an encoder network, we used NFNet L1 with efficient channel attention^57^.

To train the neural networks, we utilized datasets containing annotated objects (masks), from both leaf sides, comprising 964 instances of black feces (5.98 mm^2^), 1259 instances of damages (19.70 mm^2^), and 1718 instances of eggs. These datasets were derived from arbitrarily selected scans of leaves from 10 TSSM infested plants. Each image was partitioned into patches of size 128 × 128 pixels. Prior to being fed into the model, all images underwent standardization and a five-fold dilation process aimed at expanding the masks. To assess pixel detection efficiency, the datasets were split into training, validation, and test sets in an 80-10-10 ratio. For a conceptual, object-focused efficiency assessment, automatic and manual measurements were compared. Objects measured or counted by experts and the MITESPOTTER for efficiency calculation were excluded from training the neural network models.

### Statistical analysis

To evaluate the significance of differences (at p < 0.05) in the number of eggs laid by a female in three days and the abundance of damages and black feces between 14 *A. thaliana* ecotypes, a one-way ANOVA test was performed using R language and R Studio^58^.

### Supplementary Information

Supplementary Information.

## Data Availability

All data generated or analyzed during this study are included in this article and its supplementary information files or available from the corresponding author on reasonable request.
