# Supplementary material for: Automated imaging coupled with AI-powered analysis accelerates the assessment of plant resistance to Tetranychus urticae
Source: Sci Rep. 2024 Apr 5;14:8020. doi: 10.1038/s41598-024-58249-7 (PMC10997613; doi:10.1038/s41598-024-58249-7)
Supplement: Supplementary file 1 — Supplementary Information. [file 41598_2024_58249_MOESM1_ESM.docx]

**Supplementary material**

**Automated imaging coupled with AI-powered analysis accelerates the assessment of plant resistance to *Tetranychus urticae***

**Supplementary tables**

| **Species Variant** | **1001 Genomes ID** | **Stock Number** | **Country of Origin** |
| --- | --- | --- | --- |
| Col-0 | 6909 | CS76778 | USA |
| Cvi-0 | 6911 | CS76789 | CPV |
| Got-7 | 6921 | CS76495 | GER |
| Kondara | 6929 | CS76532 | TJK |
| Ler-0 | 7213 | CS77020 | GER |
| Ms-0 | 6938 | CS76555 | RUS |
| Rrs-7 | 7514 | CS76593 | USA |
| Tamm-2 | 6968 | CS76610 | FIN |
| Ts-1 | 6970 | CS76615 | ESP |
| Bur-0 | 7058 | CS76734 | IRL |
| Stp-0 | 9584 | CS77283 | ESP |
| Can-0 | 7063 | CS76740 | ESP |
| Car-1 | 9530 | CS76742 | ESP |
| Dor-10 | 5856 | CS76806 | SWE |

Table S1. 14 *Arabidopsis thaliana* natural accessions data

Table S2. Summary of *Arabidopsis* accessions susceptibility to *Tetranychus urticae*. We provide mean and standard error (SE) for each *A. thaliana* accession, and results of one-way ANOVA analysis for each trait: F-value, Partial Eta Squared (η_p_^2^) and p-value.

| **Accession name** | **Mean ± SE** | | | | | |
| --- | --- | --- | --- | --- | --- | --- |
|  | **Feeding symptoms area [mm^2^]** | **Relative feeding symptoms area** | **Oviposition rate** | **Relative oviposition rate** | **Feces area [mm^2^]** | **Relative feces area** |
| Car-1 | 2.32 ± 0.51 | 0.31 ± 0.07 | 15.25 ± 1.84 | 0.61 ± 0.07 | 0.13 ± 0.03 | 0.92 ± 0.20 |
| Cvi-0 | 7.27 ± 1.44 | 0.79 ± 0.13 | 32.33 ± 4.38 | 1.11 ± 0.15 | 0.15 ± 0.03 | 0.93 ± 0.15 |
| Ms-0 | 10.17 ± 3.06 | 0.92 ± 0.24 | 55.33 ± 15.70 | 2.13 ± 0.45 | 0.33 ± 0.08 | 1.93 ± 0.36 |
| Col-0 | 9.33 ± 0.68 | 1.00 ± 0.07 | 24.68 ± 1.66 | 1.00 ± 0.07 | 0.15 ± 0.01 | 1.00 ± 0.06 |
| Ts-1 | 9.76 ± 1.53 | 1.15 ± 0.27 | 39.17 ± 5.61 | 1.57 ± 0.30 | 0.17 ± 0.03 | 1.19 ± 0.23 |
| Got-7 | 18.06 ± 5.66 | 1.22 ± 0.34 | 47.5 ± 15.6 | 1.42 ± 0.30 | 0.25 ± 0.04 | 1.37 ± 0.17 |
| Bur-0 | 6.92 ± 1.83 | 1.23 ± 0.36 | 31.25 ± 8.61 | 1.39 ± 0.44 | 0.18 ± 0.03 | 1.42 ± 0.31 |
| Rrs-7 | 11.40 ± 3.05 | 1.28 ± 0.38 | 54.00 ± 6.86 | 1.78 ± 0.13 | 0.18 ± 0.04 | 1.11 ± 0.27 |
| Dor-10 | 12.16 ± 3.29 | 1.52 ± 0.35 | 15.80 ± 6.33 | 0.67 ± 0.27 | 0.16 ± 0.02 | 1.06 ± 0.22 |
| Kondara | 15.88 ± 2.26 | 1.77 ± 0.33 | 74.00 ± 20.83 | 2.41 ± 0.58 | 0.31 ± 0.06 | 1.91 ± 0.41 |
| Ler-0 | 27.82 ± 7.92 | 2.85 ± 0.54 | 71.33 ± 13.12 | 2.46 ± 0.46 | 0.34 ± 0.06 | 1.97 ± 0.32 |
| Tamm-2 | 38.50 ± 4.60 | 3.22 ± 0.73 | 104.33 ± 19.27 | 4.08 ± 0.79 | 0.32 ± 0.06 | 2.01 ± 0.47 |
| Can-0 | 15.34 ± 6.35 | 4.12 ± 1.11 | 21.25 ± 2.60 | 1.79 ± 0.73 | 1.19 ± 0.03 | 2.78 ± 0.40 |
| Stp-0 | 26.03 ± 4.64 | 4.46 ± 0.46 | 63.50 ± 11.92 | 4.84 ± 0.52 | 0.30 ± 0.05 | 2.94 ± 0.49 |
| **F-value** | 11.09 | 15.20 | 11.00 | 17.32 | 5.65 | 7.94 |
| **η_p_^2^** | 0.530 | 0.607 | 0.524 | 0.634 | 0.361 | 0.443 |
| **p-value** | <0.001 | <0.001 | <0.001 | <0.001 | <0.001 | <0.001 |

**Supplementary figures**


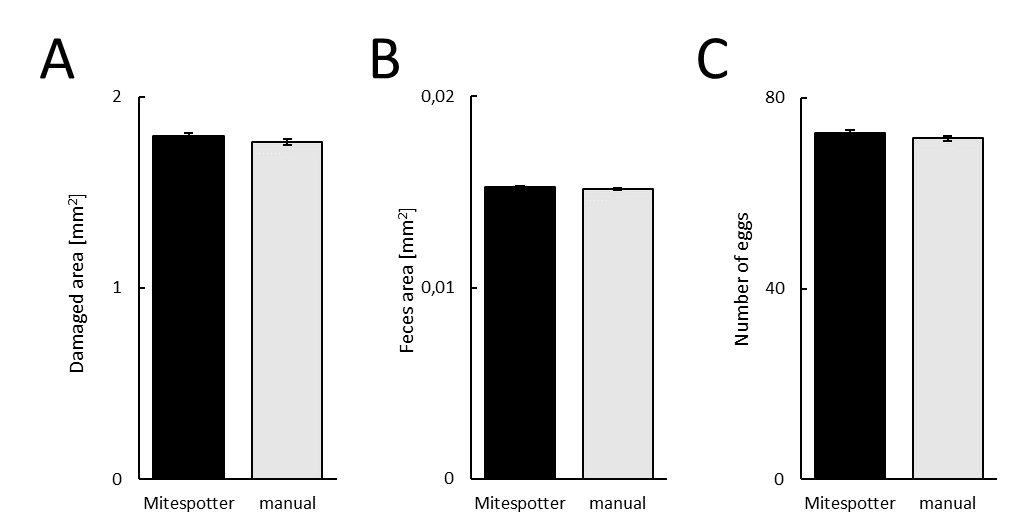
Fig. S1. Manual and Mietspotter comparison of the detected damages (**A**), feces (**B**), and eggs (**C**). To check the correctness of damages and feces detection by the Mitespotter program, the GIMP 2.10.32 program was used to manually mark the objects, and then the Fiji program was used to count pixels of the selected surface. In case of eggs detection objects were counted manually. The bars represents the means of measurements on 6 leaves (damages and feces) or plants (eggs) for Mitespotter detection (black) and manual measurement (gray). The differences in the determined damages area did not exceed 7% (the average detection accuracy was 102.87%), which results from more accurate detections made by the program. In case of area of feces, which were the most distinguishable from leaves, the average detection accuracy was 100.73% (the differences were lower than 1%). In eggs detection, the difference was not bigger than 9% and the average detection accuracy was 101.63%. No statistically significant differences were found for any of the traits using t test (*P*-value < 0.05).
